# Drinking Water

**Published:** 1886-01

**Authors:** Ella Guernsey

**Affiliations:** Sedalia‘ Mo.


### DRINKING WATER.

BY ELLA GUERNSEY.

Beware, lest in drinking deep draughts from

" The old oaken bucket that hangs in the well,"

you drink unto death.

"The Lord hath afflicted me sorely," cried Mary Nolan, a careful housewife, and the mother of six little ones, four of them lying in a darkened room at death's door. "All my life have I served Him, and tried to do my whole duty by these little ones," was her oft-repeated cry.

"My dear madame, you are not alone in your affliction ; this mysterious disease is claiming its victims daily. All that we can do is to submit to His will," said the elderly physician, who had ridden over hill and hollow in the country settlement the past twenty years, as he sat by the bedside of the little sufferers, condoling with the mother, and powerless to aid them, since calomel and quinine had failed to bring relief.

The good doctor spake truly. In twenty homes situated in a beautiful valley some member lay sick, and the way to the country cemetery was frequently travelled, and a father, mother or child laid to rest under the spring flowers and creeping myrtle.

The long, hot summer days brought no relief. Agust found Mary Nolan tearless and childless, bearing with resignation the "heavy chastening."

The more fortunate neighborhoods spoke wonderingly of the "mysterious dispensation of Providence," and lent a helping hand to the weak and sick of Pleasant Valley. The good quiet farm-people often read of foul gases, bad air and drains, and filthy water which bred disease in cities, but in the pure air and water which was so abundantly supplied Pleasant Valley, there could be no poison lurking.

A young lady, unable to bring about an investigation in any other way, bottled water from the Nolan well, a very old and moss-covered one, the stone walls of which were green with slime and moss. Slyly she sent it to a chemist in a neighboring city, who carefully analyzed it, and wrote back :

"Don't, don't drink that water! There's death in that pure, sweet water. Dig cisterns, or boil that water before using. So much the more dangerous that it is clear and cool. If 'twere brackish and warm few persons would drink of it."

But Mary Nolan, with the residents of Pleasant Valley, refused to believe that the water was impure. "It's only a crotchet of a chemist. Our fathers and grandfathers lived here and drank from these wells, and they had their seasons of affliction and health. When the weather turns cooler we shall all feel better," was the common utterance. And so they were, as less water was used without boiling, thus destroying the germs of disease lurking in it.

During the scourge of cholera, which swept away alike the rich and poor, cleanly and filthy, several years since, an ignorant colored woman living in a city built upon the banks of a beautiful river, noticed that in the country, where limestone abounded, and its inhabitants drank freely of the deliciously cool, sweet water, cholera entered into nearly every home, and was unusually violent in its attack. In her own city, those who drank much river water suffered severely. By walking two miles to the government buildings and arsenal, she could procure distilled water for family use. Daily she trudged after it, using no other, and indulging carefully in fresh vegetables and fruits during the worst of the scourge. All around her were attacked, scarcely a home escaped a visit from cholera, while this colored woman with her large family escaped the least illness.

During the summer of 1884 a lady going from New York to the West, during the long hot days, suffered severely from thirst, and an inflammation of the stomach. The hot winds swept over the prairie by night and day, and almost suffocating in the little, hot house, she drank often, and deep draughts from the well—thankful for "This one blessing; cool, sweet water." A few months found her suffering from the use of this "cool" water. A violent inflammation of delicate organs entailed a lifelong illness upon her. Not able to detect the dangerous elements in it, she was guided by her taste, and drank to her own destruction.

Water, a blessed gift to all creation, should be our first care. The "haus frau" and mother, should know to a certainty that the water and ice is free from all hurtful impurities, and then the sick ones may drink their fill without harm.

A few years since, the fever stricken patient had a sorry time of it, debarred from two necessities, fresh air and good water.

The silver pitcher, or beautifully decorated china one, does not always contain water fit for use.

In its pure state there is no lotion so healing as water, when applied to aches and external wounds.

Many children, too young to ask for it, have died for want of water, the nurse forgetting that even babies must drink, and many have died from foul water, an irritant and slow poison.

In the country, we are apt to pay little heed to the wells and drainage, relying upon the fresh air and space too much, for our own safety, and the old, old well is frequently a source of pride because of its age.

We draw water sparkling from the deep old well, display it in the crystal goblet to chance guests, thus chalenging their admiration of "our nectar," and shudder as we read of the dirt and filth prevailing in the crowded cities, wishing that the suffering ones could drink from *our* well, wondering why the man who cleaned it out last week turned so deathly sick while being let down just a few feet in our old, old well, walled with clean solid stone all grass and moss grown."

*Sedalia' Mo.*

